# Increases in the Numerical Density of GAT-1 Positive Puncta in the Barrel Cortex of Adult Mice after Fear Conditioning

**DOI:** 10.1371/journal.pone.0110493

**Published:** 2014-10-21

**Authors:** Ewa Siucinska, Adam Hamed, Malgorzata Jasinska

**Affiliations:** 1 Department of Molecular and Cellular Neurobiology, Nencki Institute of Experimental Biology, Warsaw, Poland; 2 Department of Neurochemistry, Institute of Psychiatry and Neurology, Warsaw, Poland; 3 Department of Histology, Jagiellonian University Medical College, Krakow, Poland; Tokai University, Japan

## Abstract

Three days of fear conditioning that combines tactile stimulation of a row of facial vibrissae (conditioned stimulus, CS) with a tail shock (unconditioned stimulus, UCS) expands the representation of “trained” vibrissae, which can be demonstrated by labeling with 2-deoxyglucose in layer IV of the barrel cortex. We have also shown that functional reorganization of the primary somatosensory cortex (S1) increases GABAergic markers in the hollows of “trained” barrels of the adult mouse. This study investigated how whisker-shock conditioning (CS+UCS) affected the expression of puncta of a high-affinity GABA plasma membrane transporter GAT-1 in the barrel cortex of mice 24 h after associative learning paradigm. We found that whisker-shock conditioning (CS+UCS) led to increase expression of neuronal and astroglial GAT-1 puncta in the “trained” row compared to controls: Pseudoconditioned, CS-only, UCS-only and Naïve animals. These findings suggest that fear conditioning specifically induces activation of systems regulating cellular levels of the inhibitory neurotransmitter GABA.

## Introduction

Previous work on the primary somatosensory cortex (S1, barrel cortex) has demonstrated that there is expansion of the “trained” barrels after animals acquire whisker shock conditioning [1] or whisker-trace-eyeblink conditioning [2]. There is also evidence that whisker-foot shock fear conditioning, enhances the local population response to the associated whisker stimulation in the region of barrel cortex mapping the trained whisker [3]. Learning in adult animals generates structural [4], [5], [6] functional [3] or small scale changes [7] in primary sensory cortex. Large scale changes are associated with structural and functional deficiency of the cortical circuits [8], [9].

GABA is the major inhibitory neurotransmitter in the mammalian central nervous system (CNS). GABAergic transmission is controlled in an activity-dependent manner [10], [11]. The pioneering studies of Hendry and Jones [12], [13] demonstrated activity dependent regulation of layer IV immunostained GABAergic neurons in the monkey visual cortex. The inhibitory system in the primary somatosensory cortex of rodents can also be affected by increasing peripheral stimulation [14]. Several reports have suggested that both excitatory and inhibitory circuits in the neocortex are strongly regulated by experience [15], [16], [17], [18].

The S1 GABAergic system is up-regulated when sensory stimulation is behaviorally relevant for mice. Three 10 min sessions of row B whisker-shock conditioning (CS+UCS) (CS, conditioned stimulus - tactile stimulation of a row of facial vibrissae + UCS, unconditioned stimulus - a tail shock) resulted in a rapid, transient and extinguishable expansion of 2-[^14^C] deoxy-D-glucose (2DG) labeled cortical representation of row B vibrissae. During training, only the whiskers of row B were stimulated, but training-induced 2DG labeled expansion of the cortical representation of row B vibrissae involved parts of rows A and C, on the “trained” hemisphere [1]. Interestingly, 24 hours after whisker-shock conditioning (CS+UCS), the density of GABA immunopositive cells was significantly higher in the hollows of the trained side in barrels row B and row C, but not in row A [19]. Urban-Ciecko and co-workers [20] have used whisker-shock conditioning (CS+UCS) to examine sensory learning-induced field potentials evoked in *ex vivo* slices of the barrel cortex. They found that the amplitude of responses evoked by single and repetitive stimuli in the layer IV to layer II/III pathway within the barrel column corresponding to the whisker stimulated during training was unchanged. Interestingly, in a transcolumnar pathway from the trained barrel to layer II/III of the neighboring, “untrained” column, the amplitude of responses was reduced and responses to trains of stimuli applied at 40 Hz were more quickly depressed. These findings suggest a selective weakening of excitatory transmission and/or enhancement of inhibitory transmission in transcolumnar pathways, which accompany associative learning-induced cortical plasticity. The density of GABA immunoreactive neurons in the rows of neighboring barrels A, D and E also does not change [19]. Using the same CS+UCS learning paradigm, we likewise observed an increased density of GAD67 mRNA and GAD67 neurons in the hollows of barrels representing the vibrissae activated during CS+UCS training [21], [22]. Also GAD67 immunopositive boutons are affected by whisker-shock conditioning [23]. No changes in GAD65 mRNA or protein levels were detected following the same CS+UCS learning paradigm [24]. However, how whisker-shock conditioning affects the expression of GABA transporters in the barrel B hollow is unknown.

GABA transporters (GAT-1, GAT-2, GAT-3, BGT-1) in the cerebral cortex are responsible for regulating synaptic and extrasynaptic transmitter levels in cortical circuits [25]. GAT-1 is the main high-affinity plasma membrane Na^+^/Cl^−^ dependent neuronal transporter isoform, is expressed in GABAergic neurons at/or near the synapse, and is involved in the uptake of GABA from the extracellular space into GABAergic axon terminals [25], [26],[27]. Immunocytochemical data show that GAT-1 is also expressed in non-GABAergic cells and in glia [28], [29].

The main goal of this study was to investigate how whisker-shock conditioning (CS+UCS) affects the expression of puncta of the GAT-1, in the hollows of row B barrels in “trained” hemisphere of the S1 cortex evaluated by immunocytochemistry 24 h after an associative learning paradigm. We propose the new hypothesis that whisker-shock conditioning (CS+UCS) induces activation of the trained barrels, involving an increase of GABA and GAT-1 expression 24 h later. The higher density of GAT-1 localized in plasma membrane of axon terminals and astrocytic processes of symmetric synapses results in a higher uptake of GABA and hence the elimination of GABA from the active zone in perisynaptic and extrasynaptic regions. We propose that GAT-1+ puncta specifically facilitate plasticity in the barrel B hollows in trained side 24 h after whisker-shock conditioning (CS+UCS).

In this study, we used immunocytochemistry to define of neuronal and astroglial GAT-1 puncta in CS+UCS group compared to controls that were either pseudoconditioned, CS-only, UCS-only or to Naïve animals. Data were collected using the optical disector technique [30], [31], [32], [33], which has previously been used to study a wide variety of tissues [34], [35], [23].

## Materials and Methods

### Animals

The experiments were performed on 8 week old Swiss-Webster mice (25–30 g). The animals were housed and maintained in 12/90 cages (Tecniplast, Italy) under standardized conditions with an artificial 12-hour dark/light cycle, at a constant temperature (21±2°C), 70% humidity with free access to standard food (0.25% sodium; LABOFIT B) and water. All experiments were compliant with the European Communities Council Directive of 24 November 1986 (86/609/EEC) and were approved by the Animal Care and Use Committees of the Polish Academy of Science. The protocol was approved by the First Warsaw Ethical Committee on Animal Research (Permit Number: 698/2006). All surgery was performed under sodium pentobarbital anesthesia, and all efforts were made to minimize suffering.

### Study design

The mice were given a habituation period (H) to become accustomed to a neck restraint by being placed in a restraining apparatus for 10 min a day for 21 days prior to the start of experiments. After habituation sessions, the mice were divided into the following five groups: whisker-shock conditioning (CS+UCS), pseudoconditioning (PSEUDO), whisker stimulation alone (CS-only), tail shock alone (UCS-only), NAÏVE.

In the whisker-shock conditioning (CS+UCS group) (*n* = 10; including animals for electron microscopy) mice were placed in the restraining apparatus and row B vibrissae were stimulated manually using a fine paint brush. Tactile stimulation (CS) comprised three strokes on one side of the snout. Each stroke lasted for 3 s and was applied in the posterior - anterior direction along row B of the mystacial vibrissae. Great care was taken not to touch adjacent rows of whiskers or the fur growing between the rows. In the last second of the last stroke a tail shock representing the UCS was applied (single, square, pulse 0.5 s, 0.5 mA). The electrical stimulation was discontinued simultaneously with the end of stroking. After a 6 s interval the trial was repeated. The CS+UCS stimuli were repeated four times per min, for 10 min per day, for 3 consecutive days. Animals received 120 pairings of CS+UCS trials of conditioning [1].

In the pseudoconditioning schedule (PSEUDO group *n* = 7), animals received stimulation of row B vibrissae (CS), comprised three strokes on one side of the snout. Each stroke lasted for 3 s and was applied in the posterior - anterior direction along row B of the mystacial vibrissae. Pseudoconditioning schedule allows CS to be presented regularly every 15 seconds, and UCS to be presented at random relative to CS presentation. The pseudoconditioning schedule was applied for 10 min per day, for 3 consecutive days [1].

In the whisker stimulation alone (CS-only group *n* = 7), animals received stimulation of row B vibrissae (CS), which was applied for the same duration as in the whisker-shock conditioning (CS+UCS) group over 3 days, but without a tail shock.

In tail shock alone (UCS-only group *n* = 6), the whisker stimulation described above was omitted, but a single tail shock was applied for the same duration and the same number of times as in whisker-shock conditioning (CS+UCS) group.

In a control (NAÏVE) group mice had no stimulation. Data were collected from the five right and five left hemispheres (*n* = 10).

### Behavioral responses

To evaluate the effects of habituation to a head holder, which requires 21 sessions (10 min per day), we examined head turning during the first and the last session.

In the UCS-only group, which served as an additional control, head turnings were counted during 9s immediately before delivery of the tail shock.

To evaluate the effects of training, we examined head turning in response to CS in all groups. In CS+UCS, PSEUDO, CS-only groups head turnings were counted in time during application of row B whisker stroking.

### Tissue processing

Twenty-four hours after the end of the experiments the animals were euthanized with an overdose of Nembutal (150 mg/kg i.m.) and perfused transcardially with 20 ml of 0.9% saline-heparin (5000 IU/L), followed by 150 ml of cold fixative composed of 4% paraformaldehyde (PFA) in 0.1 M phosphate–buffered saline (PBS), pH = 7.4. The brains were then removed, and postfixed in PFA for 2 h at 4°C [26]. The fixed brains were cryoprotected by treatment with 10%, 20%, and 30% sucrose solution sequentially, then frozen (−70°C) and cryosectioned tangentially to the barrel field in sections (100 µm).

### Data collection

Only sections taken from layer IV of the S1 cortex, where rows A–E were readily visible under low magnification, were used in this study. We have investigated expression of GAT-1 puncta in the hollows of “non-trained” barrels A3, C3 from the CS+UCS group, Naïve group and in the hollows of “trained” barrels B3 in each group of mice, using immunocytochemistry and stereology techniques, in order to test the hypothesis that whisker-shock conditioning specifically induces expression of the GAT-1 puncta in CS+UCS trained side hollows of row B barrels of the S1 cortex. The section thickness was measured by focusing up and down through the sections and no variation was detected: thickness and block advance (BA) were 100 µm. The BA (i) determines the hitting probability of the particles within the block, (ii) avoids deformation in the z-axis (the height) [36], and (iii) avoids mutability in the barrel area which could be related to differences in the cutting plane, and to the location in different the depth of the layer IV. The sections were stored in 0.1 M PBS at 4°C before they were processed for immunocytochemistry.

### Immunocytochemical staining

Immunocytochemical staining for GAT-1 was performed as described previously Minelli and co-workers [29]. Briefly, free floating sections were incubated overnight at 4°C in rabbit polyclonal anti-GAT-1 primary antibody (1∶1000; Chemicon, Temecula, CA in PBS), washed, then treated with biotinylated anti-rabbit IgG secondary antibody (1∶100; Vector Lab., Inc Burlingame, CA in PBS) and washed again. The ABC technique (ABC-Elite kit, Vector Lab., Inc Burlington, CA) and the DAB reaction were used to identify specific immunostaining. Sections were then rinsed in PBS, mounted on gelatin coated slides, air-dried, coverslipped, and viewed with a Nikon, Ecllipse 80i microscope.

To examine GAT-1+ staining of tangential barrel cortex sections by electron microscopy the method used was that of Minelli and co-workers [29]. Mild ethanol pretreatment was used before the immunocytochemical procedure (10%, 25%, 10%; 5 min each). The rabbit polyclonal anti-GAT-1 primary antibody (Chemicon, Temecula, CA) was diluted 1∶800. After completion of the immunocytochemical procedure as described above, sections were washed in PB, postfixed in 2.5% glutaraldehyde (30 min), washed in PB, and postfixed for 1 h in 1% OsO_4._ After dehydratation in ethanol and infiltration with Epon-Spurr resin, the sections were flat embedded between two Sigmacote (Sigma, 8F119)-coated coverslips and photographed (×5) with a Nikon Optiphot using a computer assisted Nikon DXM 1200F digital camera. The images were stacked together in Adobe Photoshop CS and the barrel field reconstructed. The B3 barrel was identified from the position of the barrels together with the characteristic pattern of blood capillaries within the barrel field. The embedded slices containing the B3 barrel were then trimmed. Small blocks, selected by inspection under light microscope, were excised, glued to blank cured epoxy, and sectioned using an ultramicrotome (Ultracut, Reichert). The ultrathin sections (60–70 nm) were lightly stained with lead citrate or left unstained and examined in a JEOL 100SX TEM electron microscope.

The specifity of primary antibody used in this study has been confirmed by company (Chemicon, Temecula, CA) of origin and have been used previously in other publications [29], [37], [38], [39], [40], [41]. As a control for the specificity of the secondary antibody binding, one section from each animal was processed according to the same protocol but omitting incubation with the anti-GAT-1 primary antibody. Controls for secondary antibody cross-reaction in mouse tissues were performed by incubating sections with a non-matching anti-species antiserum. Specific immunostaining was not observed in any of these control sections. Sections from the trained and control sides for CS+UCS group of mice were processed together.

For GAT-1+/GFAP+ double labeling, free floating tangential sections taken from layer IV of the S1 cortex were incubated overnight (at 4°C) with two primary antibodies: rabbit polyclonal anti-GAT-1 (1∶1000; Chemicon, Temecula, CA in PBS) and monoclonal anti-glial fibrillary acidic protein (GFAP clone G-A-5, CY3 conjugate 1∶800; Sigma, St. Louis, MO in PBS). After washing, the sections were incubated for 1 h at room temperature with biotinylated anti-rabbit IgG (1∶100; Vector Lab., Inc, Burlingame, CA in PBS) followed by fluorescein avidin DCS (1∶100; Vector Lab., Inc, Burlingame, CA, green fluorescence). The two antigens are separate. The limited spatial overlap (yellow) suggested that some GAT-1 is also localize to astrocytic processes. Puncta that expressed both markers GAT-1+/GFAP+ (yellow) were visualized using a Leica TCS SP2, Spectral Confocal and Multiphoton Microscope. As a control, one section from each animal was processed using the method of [42]. To confirm the specificity of the primary-secondary antibody binding, separate sections were processed as follows: (a) incubation with only one of the primary antibodies (same dilution as in the double labeled experiments; see above), followed by incubation with the mixture of secondary antisera: (b) incubation with only one of the primary antibodies followed by incubation with the non-corresponding fluorophore-conjugated secondary antiserum.

Barrels were defined according to the criteria proposed by Woolsey and Van der Loos, blood capillaries served as reference marks for each section [43]. Nuclear staining with Hoechst 33258 dye (0.5 µg/ml, Molecular Probes, Carlsbad, CA) delineated the barrel cortex prior to mounting in Vectashield Mounting Medium (Vector Lab., Inc, Burlingame, CA).

### Microscopy and stereological design

To quantify the optical density (OD), morphometry was performed on 200 individual GAT-1+ puncta. Density calibration was performed according to methods proposed by [44] by establishing that the imaging system was linear across the range of illumination intensities observed through the microscope. Images acquired via a 100x oil objective lens (Nikon, S-Plan Apo, N.A. 1.40), were and digitized by interfacing with a Retiga 2000R 12 BIT Q Imaging camera and a Leica TCS SP2 spectral confocal microscope (100x/N.A 1.4). Analysis was accomplished with computer-aided stereology-image analysis software (Image-Pro plus Version 5.0 for Windows 2000 & XP Professional, Media Cybernetics). Only the section taken from layer IV, one in the left and one in the right S1 cortex, 10–15 disector samples for each region were counted. Data were analyzed in a blind fashion with respect to the experimental condition and were scored by two different investigators. The focal depth (in µm) was measured using the microcator, where 0 µm indicates the position of the top surface of the section. Under low magnification, a contour was traced around the entire hollow of the barrel, and a sampling grid was placed over the contour in a random fashion by the computer. Each box in the grid contained a 10 µm x 10 µm counting frame representing the sampling area. The grid box dimensions represent the X and Y distances separating one counting frame from the next. Fields of vision were sampled systematically, uniformly random with X-step, the width-35.2 µm; Y-step, upward-36.4 µm; Z-axis, the height-10 µm using a motorized stage (Märzhäuser). In each field of vision, an unbiased counting frame area of 100 µm^2^ (10 µm×10 µm) was superimposed and used for sampling of the GAT-1+ puncta. It was also necessary to measure the section thickness in every single counting frame, so the computer had to calculate the disector height before the GAT-1+ puncta were sampled. The actual thickness (t) was measured by focusing all the way down to the bottom surface of the section, i.e. the point at which the last objects go out of focus. The measured t-values were recorded together with the local disector (h = 10 µm) count [36]. All GAT-1+ puncta to be counted were visualized by focusing through the disector height that came into focus at a specific focal plane. Puncta were counted if they satisfied the specific criteria [30]: (i) puncta must come into focus within the height of the optical dissector, (ii) puncta must lie entirely or partially within the counting frame or they must touch the upper and right borders (inclusion edges) and (iii) puncta must not touch the lower and left borders (exclusion edges). Counting continued until the user focused downwards past the last focal plane at the bottom of the disector, at which time the motorized stage automatically moved on to the next counting frame. Data were expressed as the average number of GAT-1+puncta (± SE). The coefficient of error (CE) is the mean of the coefficients of error of individual estimates for each group [45]. The numerical density (N_v_) estimates of GAT-1+ and GAT-1+/GFAP+ puncta per mm^3^ were calculated as follows: N_v_ = ∑ (Q^−^/a·h), where Q^−^ is the number of GAT-1 transporter puncta contained within each block of tissue recorded on the sampling grid, *a* is the area of the counting frame, and *h* is the height of the optical disector.

### Statistical analysis

The effect of habituation and tail shock alone (UCS-only) on head turning was counted from video recordings and compared between the first and the last session by paired two-tailed Student's *t*-test comparisons. Significance was accepted at the *p*<0.05 level.

In behavioral studies, the number of head turnings in response to CS during 10 min in the first and last session of each group (CS+UCS, PSEUDO, CS-only) was counted from video recordings and analyzed using the Mann-Whitney *U* test. Significance was accepted at the *p*<0.05 level.

To evaluate the effects of training we examined the numerical density (N_v_) of GAT-1+ puncta in the hollow of barrel A3 (*n* = 8) and C3 (*n* = 8) on the trained side in comparison to the hollow of barrel A3 (*n* = 8) and C3 (*n* = 8) on the control side of control hemisphere in CS+UCS by paired two-tailed Student's *t*-test comparisons. Significance was accepted at the *p*<0.05 level.

To compare differences in the numerical density (N_v_) of GAT-1+ puncta in the hollow of barrel B3 on the trained side in comparison to the hollow of barrel B3 on the control side of control hemisphere in CS+UCS group and all controls (PSEUDO, CS-only, UCS-only, and NAIVE animals), we used a two-way ANOVA (group and side treatment with repeated measures on the last factor) followed by Huynh-Feldt adjusted (H–F) *post hoc* test. The probability level *p*<0.05 was considered significant. Calculations were performed with the Graph Pad Prism version for Windows (Graph Pad Software Inc., USA) and STATISTICA 7.1. The data were expressed as means ± SE.

## Results

### Behavioral effects

In the first session of habituation animals reacted to the head holder by turning their head in all directions. In the course of habituation, the number of head turnings decreased significantly from 29.57±1.04 in the first session to 5.62±0.39 in the twenty first session (paired two-tailed Student's *t*-tests, t = 20.8; *p*<0.0001). This shows that the animals habituated to neck restraint.

In mice from the UCS-only group the number of head turnings decreased from 9.66±2.41 in the first session to 3.83±0.94 in the third session (paired two-tailed Student's *t*-test, t = 3.91; *p* = 0.011). This shows that a tail shock applied alone produced a definite observable response, i.e. reduction of head turnings.

**Figure 1:**
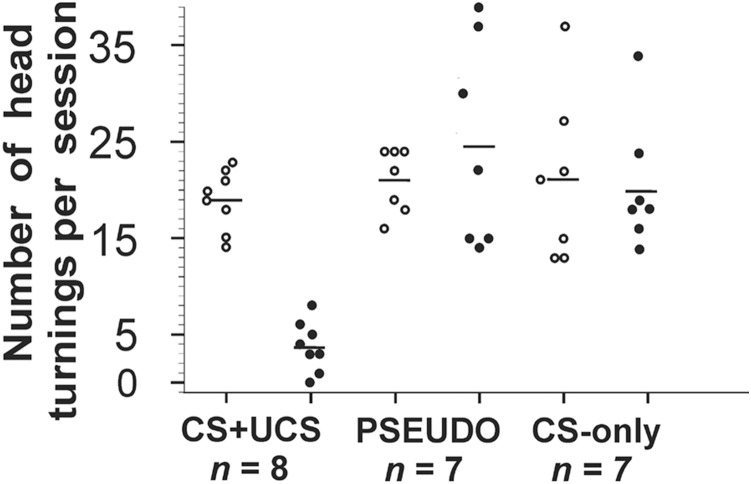
The number of head turnings per session in mice under different treatment conditions. Note that decrease of this response was observed during the last session of fear conditioning (CS+UCS *n* = 8) Mann-Whitney *U* test *p*<0.05; pseudoconditioning (PSEUDO *n* = 7), whisker stimulation alone (CS-only *n* = 7). White circle – first session, black circle - last session.

During the initial session of whisker-shock conditioning (CS+UCS), the mice often reacted to vibrissal stimulation (CS) by turning their head toward the stimulus. However, in the course of CS+UCS, the number of head turnings decreased from 19.0±1.13 in the first session to 3.75±0.92 in the third session (*p*<0.05). The decrease has not been observed in the in the case of pseudoconditioning (PSEUDO session 1, 21.0±1.25; session 3, 24.57±4.06), and in case of CS-only (CS-only session 1, 21.14±3.29; session 3, 20.42±2.54). This shows that in only during whisker-shock conditioning (CS+UCS) sessions animals learn to fear and head turning accompanied fear conditioning (Fig. 1).

### GAT-1 expression 24 h after experiments

#### Staining pattern

**Figure 2:**
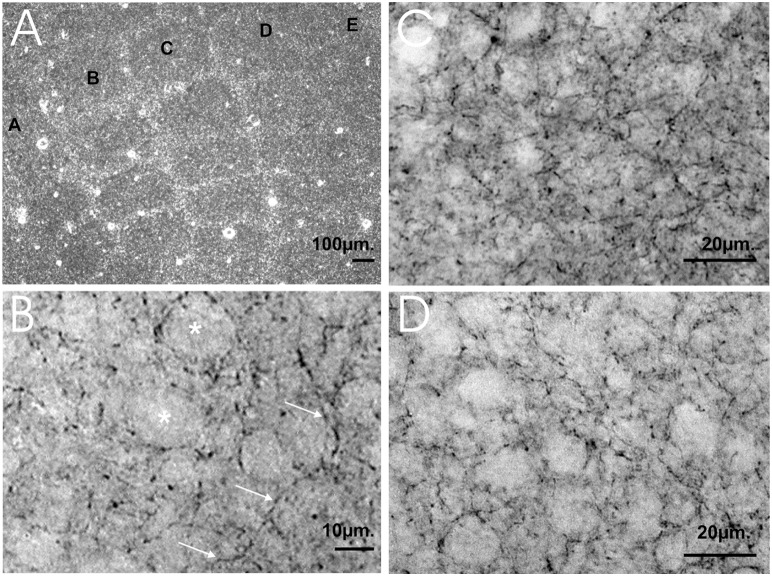
Tangential sections of the mouse barrel field immunostained for GABA transporter GAT-1. (A) An example of a tangential section of the mouse barrel field immunostained for GAT-1, letters A-E denotes rows of barrels. Scale bar 100 µm. (B) GAT-1+ puncta were observed throughout the neuropil in the barrel hollow. GAT-1+ puncta were numerous around unlabeled neuronal perikarya (asterisk). Fibers running obliquely or radially (arrowed) show irregularly spaced varicose swellings. Scale bar 10 µm. (C) High magnification micrographs from the trained side barrel B3 hollow in comparison with the control side barrel B3 hollow in the group of animals receiving CS+UCS (D). Note that CS+UCS induced an increased density of GAT-1+ puncta. Scale bar 20 µm. Immunocytochemical staining for GAT-1 was performed as described previously Minelli and co-workers [29].

The GAT-1 immunoreactivity in the S1 cortex was found in all cortical layers. The highest number of GAT-1+ puncta was in layer IV. GAT-1+ puncta were observed throughout the neuropil in the barrel hollow (Fig. 2A); they were numerous around unlabeled neuronal perikarya and also localized in fibers. GAT-1+ puncta varied in size from 0.5–1.3 µm, and in their intensity of staining from 0.3 to 0.6 optical density units (Fig. 2B).

#### GAT-1+ puncta in the barrel A3 hollows in trained and control side

After whisker-shock conditioning (CS+UCS), the average density of GAT-1+ puncta in barrel A3 hollows in the trained side (*n* = 8, 87 disectors, 629±40.57, CE 0.04), and control side (*n* = 8, 90 disectors, 665.0±26.99, CE 0.04) does not change. The N_v_ of GAT-1+ puncta barrel A3 hollows in the trained side (0.5781±0.01×10^8^/mm^3^), and control side (0.5972±0.02×10^8^/mm^3^) does not change (paired two-tailed Student's *t*-test, t = 0.6699; *p* = 0.52).

In the naïve (NAÏVE) group, we did not detect any differences in the average density of GAT-1+ puncta in the barrel A3 hollows from the left or right hemispheres. Therefore, the data (*n* = 10, 108 disectors left and right hemisphere) from these naïve controls were pooled. In the barrel A3 hollows of naïve mice the average density of GAT-1+ puncta identified was 633.8±37.31, CE 0.04 and the N_v_ was 0.582±0.03×10^8^/mm^3^ (Fig. S1).

Taken together, our data indicate that whisker-shock conditioning (CS+UCS) has no effect on expression of puncta GAT-1 in the barrel A3 hollows 24 h after associative learning paradigm.

#### GAT-1+ puncta in the barrel C3 hollows in trained and control side

After whisker-shock conditioning (CS+UCS), the average density of GAT-1+ puncta in barrel C3 hollows in the trained side (*n* = 8, 91 disectors, 711.37±43.85, CE 0.04), and control side (*n* = 8, 92 disectors, 707.37±47.47, CE 0.04) does not change. The N_v_ of GAT-1+ puncta barrel C3 hollows in the trained side (0.6244±0.019×10^8^/mm^3^), control side (0.6194±0.02×10^8^/mm^3^) does not change (paired two-tailed Student's *t*-test, t = 0.21; *p* = 0.83).

In the naïve (NAÏVE) group, we did not detect any differences in the average density of GAT-1+ puncta in the barrel C3 hollows from the left or right hemispheres. Therefore, the data (*n* = 10, 106 disectors left and right hemisphere) from these naïve controls were pooled. In the barrel C3 hollows of naïve mice the average density of GAT-1+ puncta identified was 622.8±35.06, CE 0.04 and the N_v_ was 0.587±0.02×10^8^/mm^3^ (Fig. S2).

Taken together, our data indicate that whisker-shock conditioning (CS+UCS) has no effect on expression of puncta GAT-1 in the barrel C3 hollows 24 h after associative learning paradigm.

#### GAT-1+ puncta in the barrel B3 hollows in trained and control side

**Figure 3:**
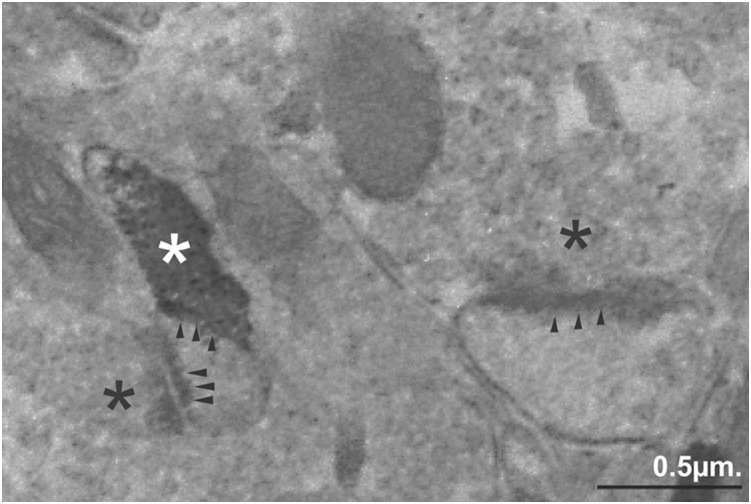
Ultrastructural localization of GAT-1 in the barrel B3 hollow in trained side CS+UCS group. GAT-1+ terminal (white asterisk), which forms a symmetrical synaptic contact (arrowheads), and the terminal (black asterisk), which ends in asymmetrical synaptic contacts (arrowheads) are localized on the same dendritic spine. The adjacent terminal (black asterisk) with asymmetric specialization (arrowheads) is unlabeled. Scale bar 0.5 µm.

After whisker-shock conditioning (CS+UCS), more GAT-1+ puncta were observed in the barrel B3 hollows in the trained side (Fig. 2C) than in the corresponding region B3 barrels on the control side (Fig. 2D). Our electron microscopic observations confirmed that GAT-1 is localized in neurons and astroglia as it has been described by [29]. In addition we found that GAT-1 immunoreactivity is also present in axon terminals forming symmetric synapses on double synapse spines (Fig. 3).

**Figure 4:**
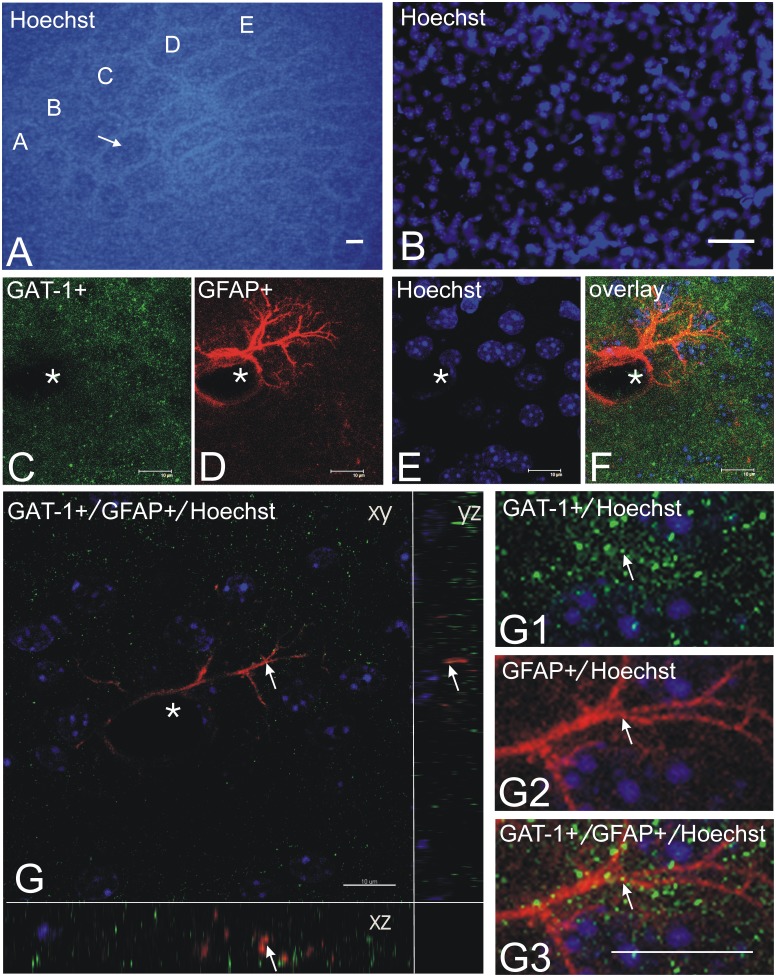
GAT-1 and GFAP in a tangential section taken from layer IV of the SI cortex. (A) Nuclear staining with Hoechst 33258 delineates the barrel cortex. Letters A–E denote rows of barrels: the arrow indicates the hollow of barrel B3. (B) Nuclear staining with Hoechst 33258 of the outline of the barrel B3 hollow. Photomicrographs C, D, E, F, depict the same field covering the hollow of barrel B3. (C) shows GAT-1 immunopositive puncta (green); (D) shows GFAP - immunopositive astrocyte (red); (E) shows nuclear staining with Hoechst 33258 dye (blue); (F) overlays images C, D, and E; (G) confocal images of immunostaining for GFAP, GAT-1 and Hoechst 33258. A GFAP+ astrocytic processes (red) contains GAT-1 (red and yellow, indicated by arrow), as in the xz and yz orthogonal views and G1–G3 higher magnification images. The images are comprised of 15 optical sections of 1000 nm thickness. White asterisks in C–G denote the same blood vessel. Scale bar: A = 100 µm, B = 20 µm, C–G = 10 µm.

Simultaneous immunodetection of GAT-1+/GFAP+ puncta in the barrel B3 hollows showed that GFAP-positive astrocyte cells express GAT-1 (Fig. 4).

We counted the number of GAT-1+ puncta localized both on neurons and on astrocytic processes. A comparison of the average number of GAT-1+ puncta in the trained side barrel B3 hollows (*n* = 8, exp side 96 disectors, 1323.9±127, CE 0.03) and in the control side barrel B3 hollows (*n* = 8, control side 96 disectors, 851±74.37, CE 0.03) showed a 54% increase in the N_v_ of GAT-1+ and a >2-fold in the N_v_ of GAT-1+/GFAP+ (N_v_ of GAT-1+ puncta in trained side barrel B3 hollows 1.089±0.04×10^8^/mm^3^ including GAT-1+/GFAP+0.0916×10^8^/mm^3^; and N_v_ of GAT-1+ puncta in control side barrel B3 hollows, 0.703±0.03×10^8^/mm^3^ including GAT-1+/GFAP+0.0384×10^8^/mm^3^).

**Figure 5:**
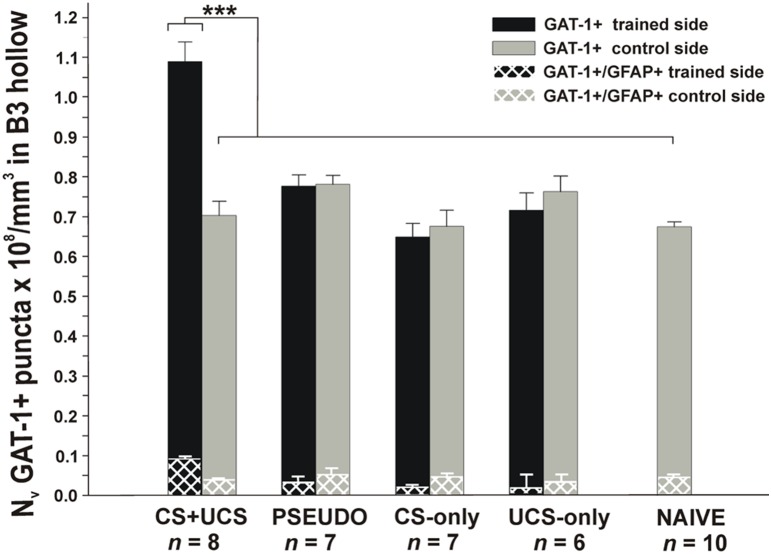
Changes in the numerical density of GAT-1+ puncta in the barrel B3 hollows in all groups. The values represent the mean numerical densities of the GAT-1+ puncta (x10^8^/mm^3^± SE. ANOVA, followed by Huynh-Feldt (H–F) *post hoc* test ***p<0.001). Whisker-shock conditioning (CS+UCS *n* = 8), pseudoconditioning (PSEUDO *n* = 7), whisker stimulation alone (CS-only *n* = 7), tail shock alone (UCS-only *n* = 6) and control (NAIVE *n* = 10). Black bars represent trained side GAT-1 expression including GAT-1+/GFAP+ (white checkered pattern) in the trained barrel B3 hollow in all group of mice. Gray bars represent control side GAT-1 expression including GAT-1+/GFAP+ (white checkered pattern) in the control barrel B3 hollow in all group of mice.

A two–way ANOVA (group and side treatment: on both the trained and control side of the brain) for numerical density (N_V_) of GAT-1+ puncta showed a significant effect of CS+UCS training [F(4,30) = 15.67, *p* = 4×10^−7^], and side treatment [F(1,30) = 7.89, *p* = 0.0086] and both factor interaction [F(4,30) = 17.25, *p* = 1×10^−7^]. A *post hoc* test for group confirmed that N_v_ of GAT-1+ puncta was higher in the CS+UCS group than in other groups (*p*<0.02). A high N_v_ of GAT-1+ puncta was found on the trained side in comparison to the control side (*post hoc* test; *p*<0.008). *Post hoc* test for group vs. side treatment interactions confirmed a significant increase of the N_v_ of GAT-1+ in the CS+UCS group in the trained side than in control side compared to all other groups: PSEUDO, CS-only, UCS-only, and Naïve (*p*<0.001) (Fig. 5 CS+UCS).

After pseudoconditioning (PSEUDO), we did not detect any differences in the average density of GAT-1+ puncta in the trained side barrel B3 hollows (*n* = 7, 85 disectors, 938.1±47.83, CE 0.03), compared with the corresponding region in the control side (*n* = 7, 84 disectors, 938.0±32.28, CE 0.03). The N_v_ of GAT-1+ puncta in the trained side barrel B3 hollows (0.7764±0.028×10^8^/mm^3^ including GAT-1+/GFAP+0.0312×10^8^/mm^3^) was not different from that in the control side barrel B3 hollows (0.7813±0.02×10^8^/mm^3^ including GAT-1+/GFAP+0.0509×10^8^/mm^3^) (Fig. 5 PSEUDO).

After whisker stimulation alone (CS-only), we did not detect any differences in the average density of GAT-1+ puncta in the trained side barrel B3 hollows (*n* = 7, 90 disectors, 832.8±66.42, CE 0.04), compared with the corresponding region in the control side (*n* = 7, 89 disectors, 858.6±68.03, CE 0.03). The N_v_ of GAT-1+ puncta in the trained side barrel B3 hollows (0.6487±0.03×10^8^/mm^3^ including GAT-1+/GFAP+0.018×10^8^/mm^3^) was not different from that in the control side barrel B3 hollows (0.6752±0.04×10^8^/mm^3^ including GAT-1+/GFAP+0.0454×10^8^/mm^3^) (Fig. 5 CS-only).

After tail shock alone (UCS-only), we did not detect any differences in the average density of GAT-1+ puncta in the barrel B3 hollows from the right side (*n* = 6, 78 disectors, 922.6±69.45, CE 0.03), compared with the corresponding region in the control side (*n* = 6, 71 disectors, 893.5±59.93, CE 0.04). The N_v_ of GAT-1+ puncta in the experimental barrel B3 hollows (0.716±0.04×10^8^/mm^3^ including GAT-1+/GFAP+0.015×10^8^/mm^3^) was not different from that in the control barrel B3 hollows (0.7631±0.03×10^8^/mm^3^ including GAT-1+/GFAP+0.0339×10^8^/mm^3^) (Fig. 5 UCS-only).

In the naïve (NAÏVE), we did not detect any differences in the average density of GAT-1+ puncta in the barrel B3 hollows from the left or right hemispheres. Therefore, the data (*n* = 10, 117 disectors left and right hemisphere) from these naïve controls were pooled. In the barrel B3 hollows of naïve mice the number of GAT-1+ puncta identified was 786±36.91, CE 0.04 and the N_v_ was 0.6738±0.012×10^8^/mm^3^ including GAT-1+/GFAP+0.043×10^8^/mm^3^ (Fig. 5 NAÏVE).

Taken together, we found that whisker-shock conditioning (CS+UCS) led to an increase in expression of neuronal and astroglial GAT-1 puncta in the trained row compared to controls: Pseudoconditioned, CS-only, UCS-only and Naïve animals. These findings suggest that fear conditioning specifically induces activation of systems regulating cellular levels of the inhibitory neurotransmitter GABA.

## Discussion

We found that in the first training session, the numbers of head turning counted during CS are higher from the numbers of head turning after habituation. Interestingly, the numbers of head turning counted before UCS in control UCS-only group are also higher from the numbers of head turning after habituation. This fact could be interpreted by that habituation to head holder stimulus has selective character, if a stimulus somewhat different from that which has been subjected to habituation is presented (CS or UCS) immediately evokes the orientation reaction.

The behavioral results presented in this work indicate that there were significantly fewer head turnings in mice from the whisker-shock conditioning (CS+UCS) group in comparison to control groups i.e. pseudoconditioned (PSEUDO) and whisker stimulation alone (CS-only). This could indicate that animals associate whisker stimulation signaling with a tail shock, an inescapable UCS stimulus. In the tail-shock alone (UCS-only) group, the whisker stimulation was omitted, but a single tail shock was applied for the same duration and the same number of times as in CS+UCS group. We found that the number of head turnings during the 9s before application of the tail-shock was significantly reduced during the subsequent trials. This possibly indicates a fear effect. For example, a reduction of movements was previously found in freezing behavior observed in fear conditioning, where foot shock applied via the wired floor of the cage was used as UCS [46].

Using the optical disector technique, we found that there was an increase in the N_v_ of GAT-1+ puncta in the barrel B hollows in trained side CS+UCS group compared to all controls. The density of GAT-1+ puncta in the barrel A and C hollows in CS+UCS group did not increase in response to the whisker-shock conditioning. The density of GAT-1+ puncta in the barrel A and C hollows in trained and control side in CS+UCS group did not reveal a difference in comparison to the density of GAT-1+ puncta in the barrel A and C hollows in the Naive animals. Therefore, our results support the hypothesis that GAT-1+ puncta specifically facilitate plasticity in the barrel B hollows in trained side 24 h after whisker-shock conditioning (CS+UCS). It has yet to be determined how GAT-1 expression accompanies fear conditioning; the following potential mechanisms should be taken under consideration.

First, GABA metabolism has been proposed as a mechanism for the control of synaptic efficacy at mammalian central inhibitory synapses [47]. The sharply increased N_v_ of GAT-1+ puncta as an effect of whisker-shock conditioning, compared to all controls, observed in the present study may be related to GABA synthesis up-regulation, as we observed increased expression of GAD67 mRNA and protein in the same CS+UCS conditioning [21], [19].

Whisker-shock conditioning (CS+UCS), in addition to increasing the transport of GABA to neurons and astrocytes, can also increase GABA turnover. There is evidence that glutamatergic synapses expressing clusters of functional postsynaptic GABA_A_ receptors in hippocampal neurons in culture are presynaptically “silent” GABA synapses [48]. Interestingly, these synapses can be “unsilenced” by loading GABA, indicating that synaptic vesicles can accommodate the usual concentration of native glutamate and a saturating concentration of GABA [48]. However, if conversion of preexisting synapses from a “silent” to an active state accompanies CS+UCS dependent up-regulation of GAT-1+ puncta, then a double-labeling study should show an increase in axon terminals GAD67 or GABA in the barrel hollows in trained side. Two particular changes have also been observed in adult mice after prolonged peripheral sensory input, namely increased GAD-IR [14] and the formation of GABAergic synapses with dendritic spines [49], [50]. In present study we found GAT-1 immunoreactivity in axon terminals forming symmetric synapses on double synapse spines. We described previously that the spines contain one asymmetrical (excitatory) and one symmetrical (inhibitory) synapse (double synapse spines), and that their density increases threefold as a result of whisker-shock conditioning with no apparent changes in the density of asymmetrical synapses. In addition, we observed the formation of new inhibitory synapses at dendrites during conditioning. An increased concentration of GABA was found in the presynaptic terminals of these synapses [51]. Therefore it seems likely that GAT-1 located in axon terminals forming symmetric synapses are associated with the fear conditioning dependent formation of new inhibitory synapses at the spines. It has recently been found that new spines are produced in layer II/III primary sensory cortical neurons to support learning during discrimination training. Both preexisting spines and newly formed spines in layer II/III neurons stabilize during perceptual learning, resulting in a net increase in spine density [52]. Recent advances in the utilizing whisker-trace-eyeblink conditioning demonstrated the timing of learning-induced neocortical spine proliferation [53].

The third potential mechanism involves GAT-1+ astrocyte puncta. Immunodetection of GAT-1 used a polyclonal antibody that is well described in the literature [29], [37], [38], [39], [40], [41]. Of special interest is the study by Ribak and co-workers, since they found that the majority of GAT-1 is distributed in smaller astroglial processes and lamellae [37]. Our data showed that whisker-shock conditioning (CS+UCS) causes an increase in the density of GAT-1+/GFAP+ puncta located in the major and middle size astroglial processes. Although, whether and how an associative learning paradigm changes the density of GAT-1+ and/or GAT-3+ puncta which were located in smaller astroglial processes and lamellae is an open question for future electron microscopy studies. Interestingly, in deep cerebellar nuclei, both GATs expressed by astrocytic processes enveloped Purkinje cell axon terminals provide a compensatory mechanism for the removal of GABA from the synaptic cleft of synapses formed by Purkinje cell axon terminals [54]. It is well known that GAT-1 is essential to the homeostatic regulation of synaptic signalling [29] and glial cells contribute to the inducing and stabilization synapses [55]. Glial modulation of glutamatergic and GABAergic synaptic transmission is documented [56]. By hindering diffusion in the extracellular space, GAT-1+ astrocytes regulate intersynaptic communication between neighboring synapses and, probably, overall volume transmission in the brain [57], [58]. Our present data suggested that whisker-shock conditioning (CS+UCS) specifically induces activation of systems regulating cellular level of the inhibitory neurotransmitter GABA as a consequence of the previously identified presynaptic increase in density of GAD67+ puncta [23], enhancement of inhibitory synaptic transmission [59], GABAergic tonic currents [60] and inhibitory synaptogenesis [51].

Finally, whether and how whisker-shock conditioning (CS+UCS) alters the concentration of other GABA transporters in the barrel cortex is only now starting to be appreciated. In this respect, it would be interesting to examine the plasticity in expression of GAT-1+ versus other GABA transporters by quantitative immunoblotting. Perhaps CS+UCS stimulates the high ambient GABA concentration [61]. There is no evidence in our data set for a specific population of GABAergic terminals contributing to the symmetrical synapses, showing an increase in expression of GAT-1 in CS+UCS group of animals. GAT-1 would be released efficiently, preventing further receptor occupancy and desensitization. Enhanced GABA_A_-R desensitization may account for the increased density of GAT-1+ puncta in the barrel B hollows trained side despite enhanced tonic GABA_A_-mediated conductance. These paths may also be similar, as the same factors that regulate phasic GABA_A_-R during training, also mediate inhibition, which regulates reuptake. Increases in tonic GABA_A_-R-mediated inhibition were reported in the hippocampus and the cerebellum of GAT-1 knock-out mice [62], [63] and following GAT-1 blockade [64], [65]. GAT-1 was found to be critical for the regulation of tonic and phasic GABA_A_-R mediated inhibition in cultured hippocampal neurons [66] and in the cerebral cortex [67]. Pairing sensory stimulation with nucleus basalis activation was found to induce increased inhibition in an activity-dependent manner to rebalance the persistent enhancement of excitation, leading to a retuned receptive field with a new preference for the paired stimulus [68]. Astrocytes may be a necessary intermediary in sensory learning-dependent modulation of inhibitory synapses in the barrel B hollows in the trained hemisphere. For example, Kang and co-workers [69] suggested that interneuronal firing elicits a GABA_B_-R-mediated elevation of calcium in surrounding astrocytes, which in turn potentiates inhibitory transmission of inhibitory synapses in the hippocampus. There is evidence that extrasynaptic GABA_B_-Rs can be activated through “leakage” of GABA from the synaptic cleft. GABA_B_-Rs are also present in synaptic terminals [70]. Furthermore, Davies and Collingridge [71] demonstrated that a process initiated by the activation of GABA_B_ auto receptors provides the dynamic changes in synaptic inhibition.

## Supporting Information

Figure S1 **Changes in the numerical density of GAT-1+ puncta in the barrel A3 hollows in CS+UCS and NAIVE groups.** The numerical density (N_v_) of GAT-1+ puncta in the barrel A3 hollow in trained side in comparison with barrel A3 hollow in the control side in the group of animals receiving whisker-shock conditioning (CS+UCS *n* = 8) and naive control (NAIVE *n* = 10). Black bars represent GAT-1 expression in the barrel A3 hollow in “trained” side. Gray bars represent GAT-1 expression in the barrel A3 hollow in control side. Data are expressed as mean ± SE. No significant differences were found between the groups.
(TIF)

Figure S2 **Changes in the numerical density of GAT-1+ puncta in the barrel C3 hollows in CS+UCS and NAIVE groups.** The numerical density (N_v_) of GAT-1+ puncta in the barrel C3 hollow in trained side in comparison with the barrel C3 hollow in the control side in the group of animals receiving whisker-shock conditioning (CS+UCS *n* = 8) and naive control (NAIVE *n* = 10). Black bars represent GAT-1 expression in the barrel C3 hollow in trained side. Gray bars represent GAT-1 expression in the barrel C3 hollow in control side. Data are expressed as mean ± SE. No significant differences were found between the groups.
(TIF)
