# Supplementary figures and images for: Increases in the Numerical Density of GAT-1 Positive Puncta in the Barrel Cortex of Adult Mice after Fear Conditioning
Source: PLoS One. 2014 Oct 21;9(10):e110493. doi: 10.1371/journal.pone.0110493 (PMC4204871; doi:10.1371/journal.pone.0110493)

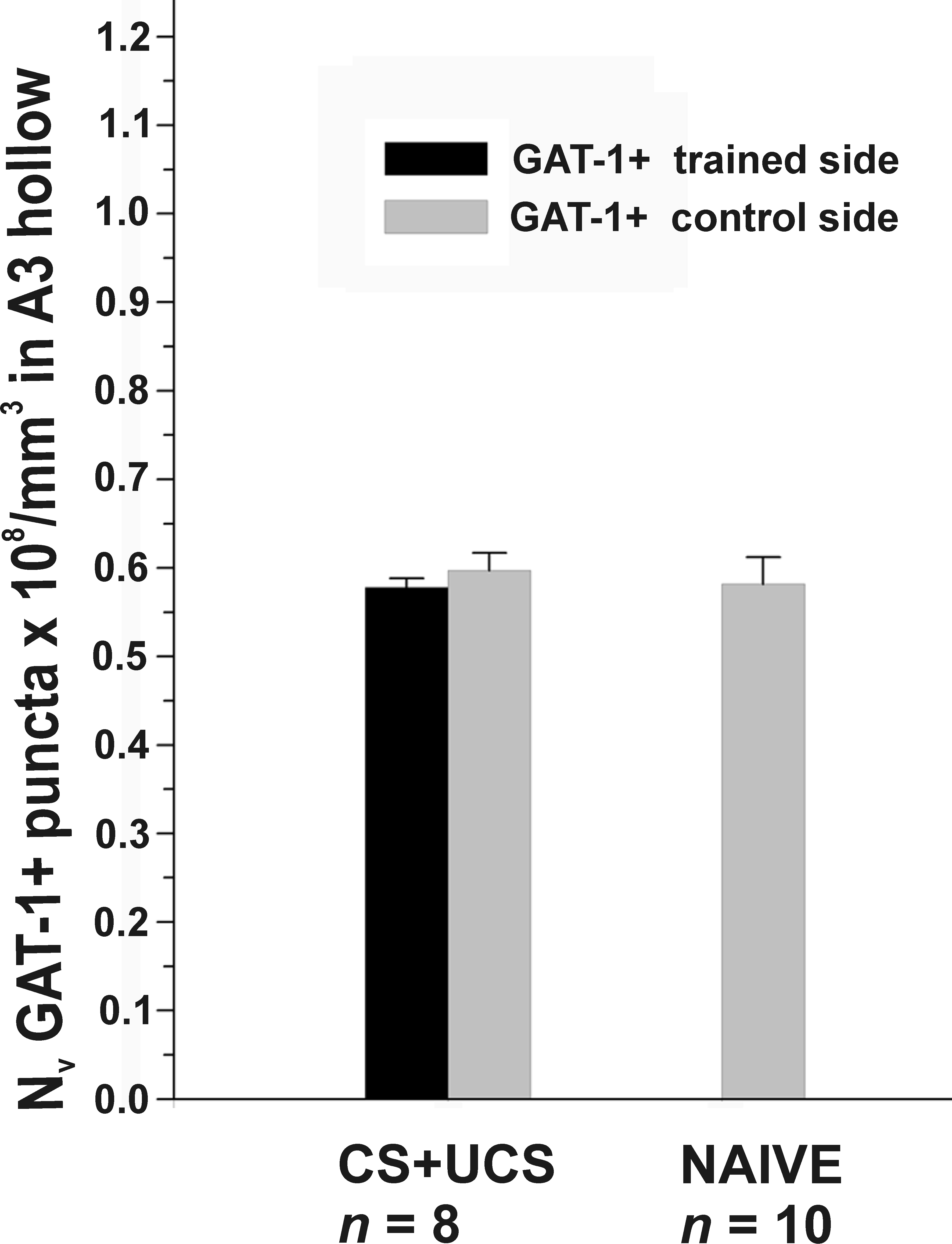

Supplement: Figure S1 — Changes in the numerical density of GAT-1+ puncta in the barrel A3 hollows in CS+UCS and NAIVE groups. The numerical density (Nv) of GAT-1+ puncta in the barrel A3 hollow in trained side in comparison with barrel A3 hollow in the control side in the group of animals receiving whisker-shock conditioning (CS+UCS n = 8) and naive control (NAIVE n = 10). Black bars represent GAT-1 expression in the barrel A3 hollow in “trained” side. Gray bars represent GAT-1 expression in the barrel A3 hollow in control side. Data are expressed as mean ± SE. No significant differences were found between the groups. (TIF) [file pone.0110493.s001.tif]

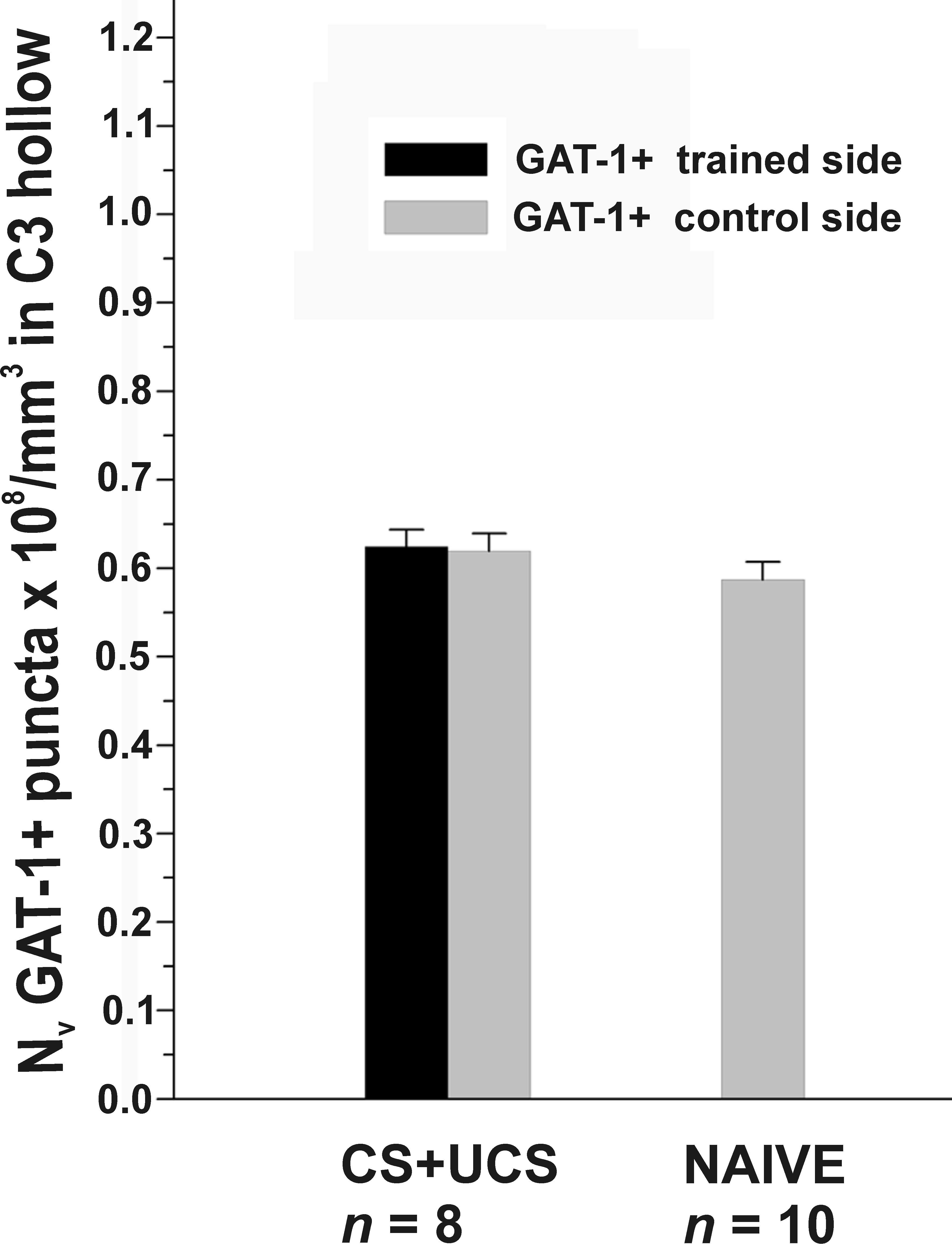

Supplement: Figure S2 — Changes in the numerical density of GAT-1+ puncta in the barrel C3 hollows in CS+UCS and NAIVE groups. The numerical density (Nv) of GAT-1+ puncta in the barrel C3 hollow in trained side in comparison with the barrel C3 hollow in the control side in the group of animals receiving whisker-shock conditioning (CS+UCS n = 8) and naive control (NAIVE n = 10). Black bars represent GAT-1 expression in the barrel C3 hollow in trained side. Gray bars represent GAT-1 expression in the barrel C3 hollow in control side. Data are expressed as mean ± SE. No significant differences were found between the groups. (TIF) [file pone.0110493.s002.tif]
